# Temporal Trends of Hyponatremia in Patients with Respiratory and Intrathoracic Cancers Treated with Chemotherapy and Immune Checkpoint Inhibitors

**DOI:** 10.3390/cancers17091459

**Published:** 2025-04-26

**Authors:** Kuo-Cheng Lu, Ching-Liang Ho, Joshua Wang, Cai-Mei Zheng, Kuo-Wang Tsai, Yi-Chou Hou, Chien-Lin Lu

**Affiliations:** 1 Division of Nephrology, Department of Medicine, Taipei Tzu Chi Hospital, Buddhist Tzu Chi Medical Foundation, New Taipei City 23142, Taiwan; tch33730@tzuchi.com.tw; 2 Division of Hematology and Oncology, Taipei Tzu Chi Hospital, Buddhist Tzu Chi Medical Foundation, New Taipei City 23142, Taiwan; hochingliang@yahoo.com.tw; 3 Department of Research, Taipei Tzu Chi Hospital, Buddhist Tzu Chi Medical Foundation, New Taipei City 23142, Taiwan; j3.reilly@qut.edu.au; 4 School of Biomedical Sciences, Queensland University of Technology, Brisbane, QLD 4001, Australia; 5 Division of Nephrology, Department of Internal Medicine, Shuang Ho Hospital, School of Medicine, College of Medicine, Taipei Medical University, New Taipei City 11031, Taiwan; 11044@s.tmu.edu.tw; 6 TMU Research Center of Urology and Kidney, Taipei Medical University, New Taipei City 11031, Taiwan; 7 Division of Nephrology, Department of Internal Medicine, Cardinal-Tien Hospital, School of Medicine, College of Medicine, Fu Jen Catholic University, New Taipei City 24205, Taiwan; tch33225@tzuchi.com.tw; 8 School of Medicine, College of Medicine, Fu Jen Catholic University, New Taipei City 24205, Taiwan; 127097@mail.fju.edu.tw; 9 Division of Nephrology, Department of Internal Medicine, Fu Jen Catholic University Hospital, Fu Jen Catholic University, New Taipei City 24352, Taiwan

**Keywords:** cisplatin, hyponatremia, immune checkpoint inhibitor, temporal trends

## Abstract

Background: Immune checkpoint inhibitors (ICIs) offer a novel approach to cancer treatment by enhancing immune responses against malignant cells. However, ICIs are associated with immune-related adverse events (irAEs), including hyponatremia, a potentially severe electrolyte disturbance. The risk of hyponatremia increases further when ICIs are combined with cisplatin, a nephrotoxic chemotherapy agent widely used in treating respiratory and intrathoracic cancers. This study investigated the incidence, severity, and temporal dynamics of hyponatremia in patients treated with ICIs alone or in combination with cisplatin. Methods: A retrospective cohort study was conducted using data from the TriNetX global health research network. Patients with respiratory or intrathoracic malignancies (*n* = 14,026) were divided into two groups: ICI-only (*n* = 7013) and ICI with cisplatin combination (*n* = 7013), matched using propensity scores. Hyponatremia was categorized into mild (130–134 mmol/L), moderate (125–129 mmol/L), and severe (<125 mmol/L). Temporal trends and cumulative incidence over 90 days were analyzed using Poisson regression. Results: The combination group exhibited a higher cumulative incidence of hyponatremia across all severity levels, with early-phase risk peaking within 20 days of treatment. Rate ratios for mild, moderate, and severe hyponatremia were significantly elevated in the combination group (*p* < 0.01). Conclusions: Hyponatremia is a significant complication in patients receiving ICIs, particularly when combined with cisplatin. Early monitoring and tailored management are essential to mitigate risks and optimize treatment outcomes.

## Simple Summary

Cancer therapies combining immune checkpoint inhibitors (ICIs) with chemotherapy drugs such as cisplatin significantly improve patient outcomes. However, this combination therapy increases the risk of developing low sodium levels in the blood (hyponatremia), especially within the first month of treatment. Hyponatremia may cause symptoms ranging from mild confusion and fatigue to severe complications such as seizures or coma if not promptly managed. By analyzing data from patients with respiratory and intrathoracic cancers, we found that those treated with both ICIs and cisplatin were more likely to develop hyponatremia early during therapy. The regular monitoring of sodium levels and early identification of at-risk patients are crucial to enhance safety, prevent severe complications, and optimize treatment outcomes.

## 1. Introduction

Immune checkpoint inhibitors (ICI) offer a novel therapeutic strategy for various cancers by strengthening the immunological response against malignant cells. These monoclonal antibodies enhance T-cell activation and improve tumor suppression through disinhibiting immune response signaling components such as programmed cell death protein 1 (PD-1), programmed death-ligand 1 (PD-L1), and cytotoxic T-lymphocyte associated protein 4 (CTLA-4) [1]. Since their introduction, ICIs have become a cornerstone in treating diverse cancers, including lung cancer, renal cell carcinoma, melanoma, and lymphoma, significantly extending patient survival [2,3,4].

Despite their groundbreaking efficacy, the rising use of ICIs has revealed a spectrum of immune-related adverse events (irAEs), which range from mild inflammatory responses to severe systemic complications. Among these, electrolyte disturbances, particularly hyponatremia, have attracted increasing attention [5]. Hyponatremia, defined as a serum sodium level below 135 mmol/L, is commonly observed in cancer patients and has been linked to poorer clinical outcomes [6,7,8]. Emerging evidence indicates that low serum sodium levels, both at the baseline and during treatment, are also associated with significantly impaired survival in patients undergoing ICI therapy [9].

The clinical significance of hyponatremia extends beyond its frequency. Symptoms vary depending on severity, ranging from nonspecific signs such as dizziness, nausea, and fatigue in mild cases to confusion and gait disturbances in moderate cases. Severe hyponatremia can lead to seizures, coma, and even death. Understanding these symptom patterns is essential for timely diagnosis and appropriate management, particularly in oncology patients where electrolyte imbalances can rapidly worsen outcomes [10].

The risks associated with ICIs become more complex when combined with nephrotoxic chemotherapeutic agents such as cisplatin. Cisplatin, widely used in solid tumor treatments, is known for its nephrotoxic effects including renal tubular damage and electrolyte imbalances [11]. While the combination of ICI and cisplatin has demonstrated enhanced antitumor efficacy, it also amplifies the risk of renal and endocrine toxicities including hyponatremia—a potentially severe but manageable complication.

Respiratory and intrathoracic cancers, such as small cell lung cancer (SCLC) and non-small cell lung cancer (NSCLC), are particularly vulnerable to hyponatremia. This complication is reported in 25–44% of SCLC patients and approximately 27% of NSCLC patients, largely attributed to paraneoplastic syndromes like the syndrome of inappropriate antidiuretic hormone secretion (SIADH), making hyponatremia a frequent and clinically significant issue in these populations [7,12,13,14]. Additionally, ICIs themselves have been implicated as a contributing factor. A retrospective analysis found that 62% of patients receiving ICIs experienced hyponatremia, with 6% suffering from severe forms (serum sodium <124 mEq/L).

Given the prevalence of hyponatremia in respiratory and intrathoracic cancers as well as its association with ICI therapy, there is a pressing need to understand the temporal trends and cumulative risks of this complication. This study sought to address these gaps by investigating hyponatremia incidence and progression in patients treated with ICIs alone or in combination with cisplatin, providing actionable insights for optimizing cancer treatment and patient safety.

## 2. Materials and Methods

### 2.1. Study Design and Data Source

This retrospective cohort study was conducted using de-identified patient data obtained from the TriNetX Global Collaborative Network, which includes real-time electronic health records from 143 participating healthcare organizations. All data used in this analysis met the international deidentification standards including those outlined in the Health Insurance Portability and Accountability Act (HIPAA). The study received ethics approval from the Institutional Review Board of Taipei Tzu Chi Hospital (Approval Number: 13-IRB141). Data retrieval was performed on 7 January 2025, according to the predefined inclusion and exclusion criteria (Supplementary Data S1). The study was conducted in accordance with the Strengthening the Reporting of Observational Studies in Epidemiology (STROBE) guidelines (Supplementary Data S2).

### 2.2. Study Cohorts

Eligible participants were adults aged 18 years or older diagnosed with malignant neoplasms of the respiratory and intrathoracic organs (ICD-10-CM: C30–C39) between 1 January 2011 and 1 January 2021. Individuals with a diagnosis of primary adrenocortical insufficiency (ICD-10-CM: E27.1) were excluded due to its potential influence on sodium regulation. Two treatment groups were analyzed: the first group included 7013 patients treated exclusively with ICI, such as durvalumab, avelumab, ipilimumab, tremelimumab, pembrolizumab, nivolumab, atezolizumab, and cemiplimab, without prior exposure to cisplatin or carboplatin, and the second group consisted of 14,782 patients receiving a combination of ICI (same drugs as listed) with cisplatin or carboplatin.

To provide a clearer understanding of the therapeutic regimens, a supplementary table (Supplementary Data S3) outlines the additional chemotherapy agents often administered alongside ICI or in combination with cisplatin/carboplatin in both cohorts. This supplementary data emphasizes the heterogeneity and complexity of treatment strategies within the study population.

### 2.3. Index Date and Follow-Up Period

The index date was defined as the earliest date on which all inclusion criteria specific to each cohort were satisfied. Follow-up for outcome assessment commenced one day after the index date and extended through 180 days post-index. To ensure temporal validity, patients with documented outcomes prior to the index date were excluded from the corresponding outcome-specific analyses. This approach minimized the risk of reverse causation and allowed for a clearer attribution of events to post-treatment exposures.

### 2.4. Data Analysis

**Figure 1:**
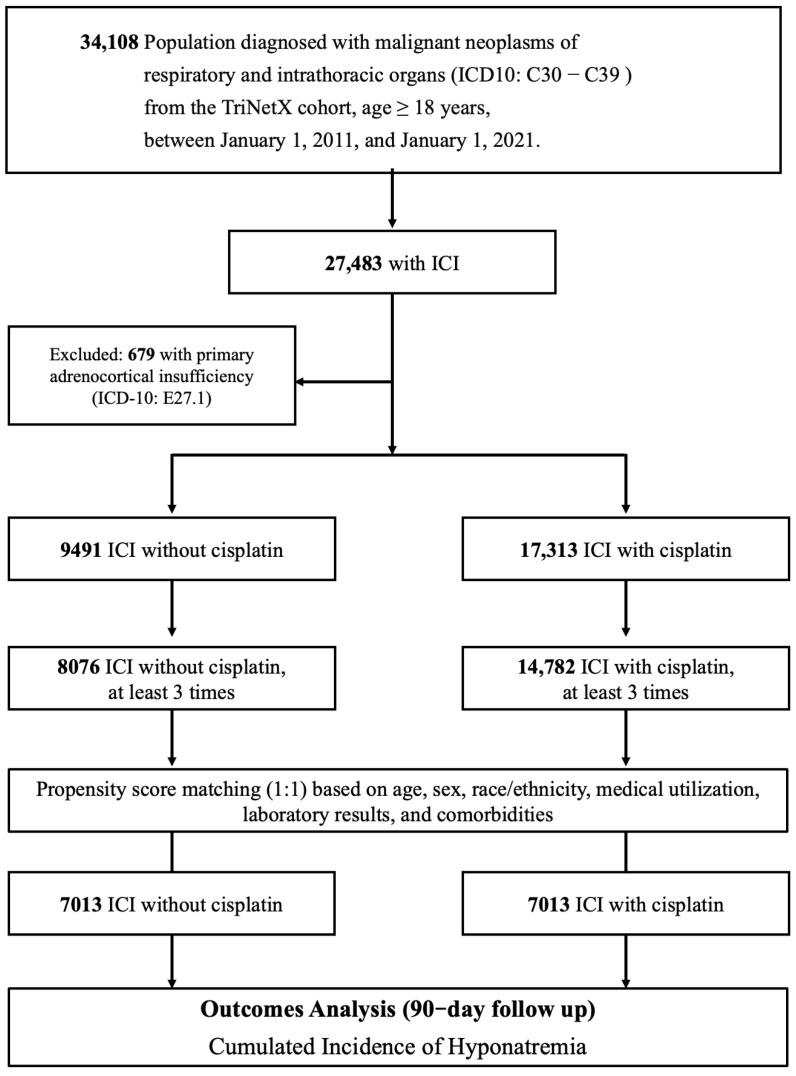
Algorithm for patient selection and enrollment in the study. This figure outlines the stepwise process used for patient selection and enrollment in the study. ICI: immune checkpoint inhibitor, Cis: cisplatin or carboplatin.

To reduce potential bias and enhance comparability, we applied 1:1 propensity score matching using a nearest-neighbor method with a caliper of 0.1 pooled standard deviations. Matching variables included key demographic factors (e.g., age, sex), comorbid conditions (e.g., diabetes, hypertension), and baseline laboratory measures (e.g., serum sodium and creatinine levels) up to one month prior to all inclusion criteria being met (Table 1). Post-matching, a total of 7013 patients remained in each group, allowing for a balanced comparative analysis (Figure 1). Detailed pre- and post-matching baseline characteristics are provided in Supplementary Data S4.

Outcomes were assessed over a six-month follow-up period, starting one day after the index date. Descriptive statistics were used to summarize the baseline demographic and clinical parameters, with standardized differences applied to ensure balance post-matching and mitigate the confounding effects. During a 90-day follow-up period, beginning one day after the index date, the incidence of hyponatremia was evaluated. Hyponatremia was stratified by severity using the widely accepted serum sodium thresholds: values between 130 and 134 mmol/L were defined as mild, 125 to 129 mmol/L as moderate, and less than 125 mmol/L as severe. These cut-offs align with classifications reported in prior clinical research and guidelines [15,16,17]. The cumulative incidence of hyponatremia (allowing for repeated measures from the same patient) was analyzed using Poisson regression modeling to identify statistically significant differences between the ICI with cisplatin combination group and the ICI without cisplatin group. Poisson regression modeling was used due to its robustness when analyzing datasets with repeated measurement [18]. Time-dependent event incidences were examined at 10-day intervals to uncover detailed temporal risk patterns, particularly during the early treatment phase. To account for potential differences in monitoring the intensity and serum sodium testing frequency, the daily number of tests was recorded. Normalized rates of positive tests were calculated for each severity level of hyponatremia, facilitating a more nuanced understanding of how monitoring variations might influence the observed risk patterns over time.

## 3. Results

**Figure 2:**
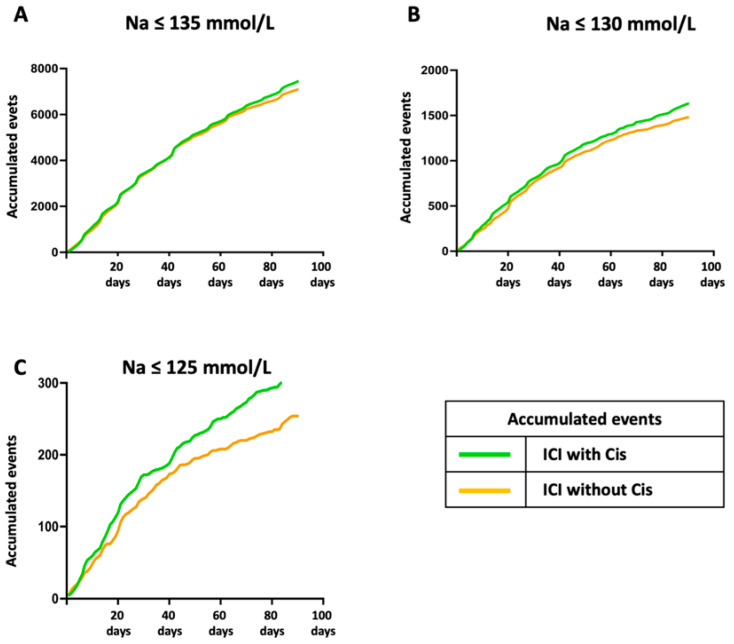
Daily cumulative events of hyponatremia over 90 days across sodium thresholds. Over the 90-day observation period, the daily cumulative number of hyponatremia events was tracked for three sodium concentration thresholds. (**A**) For Na ≤ 135 mmol/L, the ICI with cisplatin combination group reported 2190 cumulative events by Day 20 and 7437 by Day 90 compared with 2139 and 7092 events in the ICI group, respectively. (**B**) For Na ≤ 130 mmol/L, the ICI with cisplatin combination group reached 542 events by Day 20 and 1631 by Day 90, while the ICI group recorded 473 and 1481 events. (**C**) For Na ≤ 125 mmol/L, the cumulative events in the ICI with cisplatin combination group reached 119 by Day 20 and 323 by Day 90, compared with 94 and 254 events in the ICI group. ICI: immune checkpoint inhibitor, Cis: cisplatin or carboplatin.

The temporal analysis of hyponatremia over the 90-day observation period revealed distinct trends across the sodium thresholds and treatment groups. As depicted in Figure 2, the cumulative incidence of hyponatremia demonstrated a consistent increase over time for both mild and moderate cases, with the ICI with cisplatin combination group showing a slightly elevated risk compared with the ICI without cisplatin group. The divergence between the two groups became more apparent with increasing severity, particularly in severe hyponatremia, where the ICI with cisplatin combination group exhibited a notable rise in cumulative events.

Poisson regression modeling was utilized to assess whether the difference in accumulated hyponatremia instances between treatment groups was significant. The ICI with cisplatin combination group consistently exhibited significantly higher rates of severe (rate ratio = 1.272, *p* = 0.00416), moderate (rate ratio = 1.101, *p* = 0.00719), and mild hyponatremia (rate ratio = 1.049, *p* = 0.00421).

**Figure 3:**
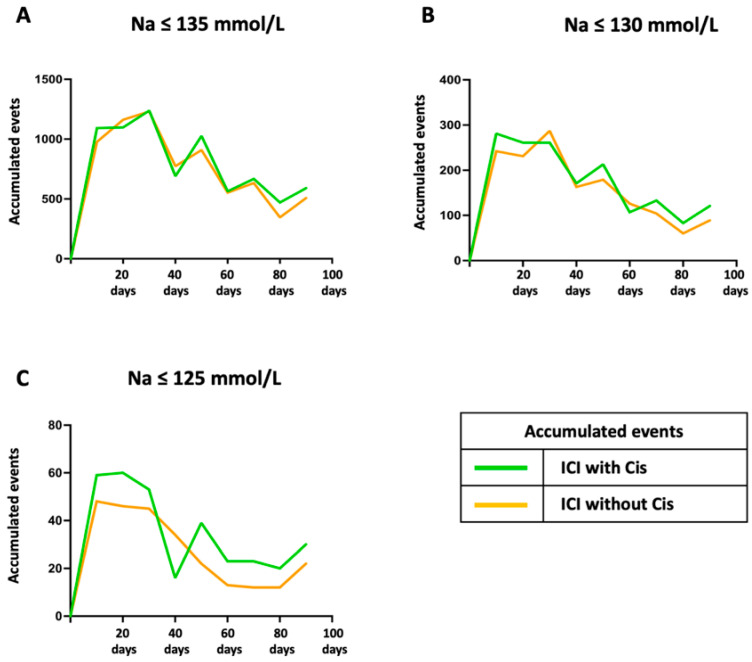
Accumulated hyponatremia events over 90 days in 10-day intervals across different sodium thresholds. Accumulated hyponatremia events across a 90-day observation period, depicted in 10-day intervals, demonstrated differences between patients treated with ICI combined with cisplatin (green lines) and those treated without cisplatin (orange lines). Panel (**A**) (Na ≤ 135 mmol/L) shows a largely comparable mild hyponatremia event accumulation between the two groups, with minimal variation throughout the observation period. Panel (**B**) (Na ≤ 130 mmol/L) reflects moderate hyponatremia trends, where slight differences emerged around Days 20 and 40, with the ICI and cisplatin combination group consistently displaying a marginally higher cumulative incidence. Panel (**C**) (Na ≤ 125 mmol/L) highlights the most significant disparity in severe hyponatremia, particularly within the first 20 days, where the combination cohort experienced a notably higher accumulation of events. ICI: immune checkpoint inhibitor, Cis: cisplatin or carboplatin.

Further stratification of the data in 10-day intervals, as shown in Figure 3, provided a more granular view of these patterns. While mild hyponatremia displayed parallel trends between the two groups, moderate and severe cases revealed more pronounced differences during the early phase of treatment. The ICI with cisplatin combination group consistently displayed a higher accumulation of events, particularly within the first 20 days, underscoring the heightened risk during the initial treatment phase.

**Figure 4:**
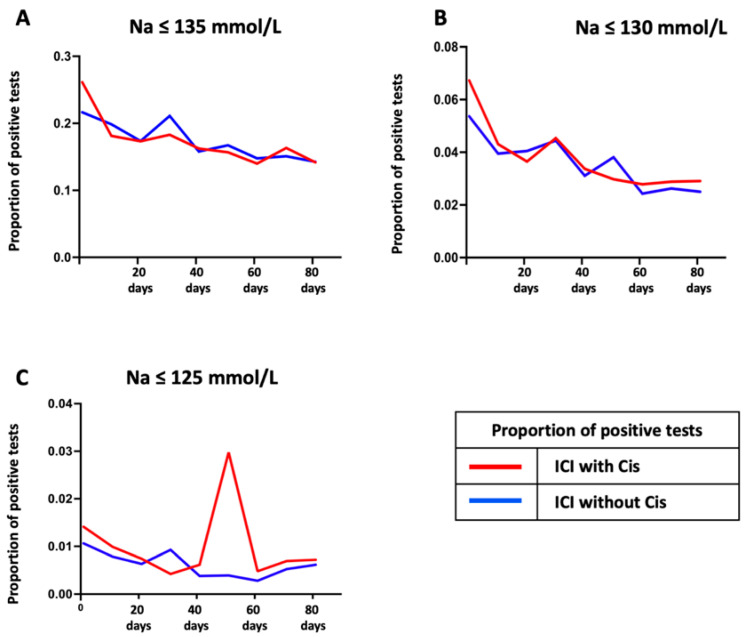
Proportion of positive hyponatremia tests over 90 days across sodium thresholds. The proportions of daily positive hyponatremia tests are shown for patients treated with ICI and in combination with cisplatin over a 90-day observation period. Sodium thresholds assessed include (**A**) Na ≤ 135 mmol/L, (**B**) Na ≤ 130 mmol/L, and (**C**) Na ≤ 125 mmol/L. In (**A**), the positive test proportions peaked within the initial 20 days and gradually declined, stabilizing after Day 60. The ICI with cisplatin combination group showed marginally higher proportions than the ICI group beyond Day 40. In (**B**), similar declining trends were observed for Na ≤ 130 mmol/L, with minimal differences between the two groups throughout the observation period. Notably, (**C**) demonstrated a sharp peak in positive tests for Na ≤ 125 mmol/L between Days 51 and 60 in the ICI with cisplatin group, while the ICI without cisplatin combination group remained relatively stable. ICI: immune checkpoint inhibitor, Cis: cisplatin or carboplatin.

The proportions of positive hyponatremia tests over time, s illustrated in Figure 4, highlighted temporal fluctuations in risk. Mild and moderate hyponatremia showed comparable declining trends in both groups, stabilizing after the initial 20 days. However, for severe hyponatremia, the ICI without cisplatin combination group exhibited a relatively stable pattern, while the ICI with cisplatin group experienced a distinct peak in positive tests during the mid-treatment period.

## 4. Discussion

This study highlights notable differences in the incidence and temporal dynamics of hyponatremia between patients treated with ICI without cisplatin and those receiving ICI with cisplatin combination therapy. Over the 90-day observation period, the ICI with cisplatin combination group demonstrated consistently higher cumulative incidences of hyponatremia across all sodium thresholds (Na ≤ 135 mmol/L, ≤130 mmol/L, and ≤125 mmol/L), with statistically significant differences observed for mild, moderate, and severe hyponatremia. Temporal trends further revealed that hyponatremia incidence peaked within the initial 30 days of treatment, with the ICI with cisplatin combination group displaying a higher early risk, particularly for moderate and severe hyponatremia. The normalized rates of positive hyponatremia tests also indicated distinct patterns, with the ICI without cisplatin combination group showing stable risk for severe cases while the ICI with cisplatin group exhibited a mid-treatment peak. Together, these results emphasize the heightened risk of hyponatremia during the early treatment phase and the need for proactive monitoring, particularly in patients receiving ICI with cisplatin combination therapy.

Hyponatremia has been observed in up to 62% of patients treated with ICI, with severe cases (Na ≤ 124 mEq/L) occurring in approximately 6%, typically around 164 days after treatment initiation [19]. Cisplatin is associated with renal salt-wasting syndrome (RSWS), affecting 1–10% of patients. RSWS-related symptoms, including hyponatremia and hypovolemia, usually manifest within 12 h to one month post-treatment and often require aggressive salt and water replacement for recovery over 3 days to 3 weeks [20]. The combination of ICI and cisplatin introduces overlapping timelines of hyponatremia risk: ICI-induced hyponatremia develops gradually due to irAEs, while cisplatin-related RSWS occurs acutely within days or weeks. This overlap likely accounts for the heightened early-phase risk observed in our study and underscores the importance of timely diagnosis, close monitoring, and personalized management.

Mechanistically, hyponatremia in this setting is multifactorial and likely synergistic. ICIs may cause immune-related endocrine disturbances, such as adrenalitis or hypophysitis, leading to secondary adrenal insufficiency, impaired cortisol production, and compromised water clearance. SIADH, characterized by inappropriate ADH secretion, is another well-documented immune-related adverse event [21,22,23,24]. Concurrently, cisplatin exerts direct nephrotoxic effects on renal tubules, resulting in natriuresis, volume contraction, and secondary ADH elevation [25,26,27]. These mechanisms may be further amplified by the co-administration of other chemotherapeutics [28,29] as well as nonspecific stressors such as chemotherapy-related nausea, pain, or systemic inflammation [30,31].

Importantly, the pathophysiology of ICI-induced hyponatremia differs by checkpoint target. CTLA-4 inhibitors like ipilimumab are more commonly associated with hypophysitis and subsequent adrenal insufficiency, leading to cortisol deficiency and impaired water excretion [32,33]. In contrast, PD-1 and PD-L1 inhibitors (e.g., nivolumab, pembrolizumab) are more frequently linked to SIADH-like presentations, likely due to cytokine-mediated ADH dysregulation [34,35]. Recognizing these mechanistic distinctions is essential for tailoring diagnostic and therapeutic approaches based on the ICI subclass.

Beyond the direct drug effects, several patient- and cancer-related factors may predispose individuals to ICI-associated hyponatremia. These include autoimmune diseases (e.g., hypothyroidism, adrenalitis), acute kidney injury, and the concomitant use of sodium-lowering medications such as thiazide diuretics or SSRIs [19,36,37,38]. Cancer-related factors such as ectopic ADH production and high tumor burden may further contribute to paraneoplastic hyponatremia [39]. Although our propensity score matching accounted for major comorbidities like diabetes and hypertension, more granular data—particularly regarding autoimmune status and tumor biology—were not available, limiting our ability to control for these potential confounders.

In addition, several commonly prescribed medications in oncology practice, such as corticosteroids, antibiotics, and proton pump inhibitors, may modulate immune responses or alter the gut microbiota, which could in turn affect the efficacy and toxicity of immune checkpoint inhibitors [40]. These agents have been implicated in modifying the risk of immune-related adverse events, including hyponatremia, through mechanisms such as the exacerbation of electrolyte imbalances or an induction of systemic inflammation via microbiome disruption. Although detailed medication histories were not available in our dataset, this highlights the need to consider potential pharmacologic interactions when managing patients undergoing ICI-based therapy.

Furthermore, other anticancer agents like cetuximab and vinorelbine have also been implicated in hyponatremia. Cetuximab, an anti-EGFR monoclonal antibody, induces SIADH through systemic inflammatory effects, while vinorelbine, a vinca alkaloid, disrupts the hypothalamic–pituitary axis [41,42]. These findings suggest that hyponatremia risk extends beyond specific regimens, warranting comprehensive risk management across oncology therapies.

From a clinical perspective, our findings support the implementation of routine sodium monitoring, particularly within the first month of therapy. Management strategies should be tailored to the underlying etiology: SIADH may respond to fluid restriction or vasopressin receptor antagonists such as tolvaptan, whereas adrenal insufficiency-related hyponatremia may require corticosteroid supplementation [43,44]. For RSWS induced by cisplatin, volume and electrolyte repletion is critical to prevent severe complications [27,45]. A balanced assessment of therapeutic benefits versus hyponatremia risk is crucial, especially during the vulnerable early treatment phase.

Several limitations should be acknowledged. First, due to the nature of the TriNetX platform, individual-level data—including symptom documentation—were inaccessible. Moreover, many symptoms associated with hyponatremia (e.g., confusion, fatigue, nausea, or seizures) are not reliably captured using ICD-10 codes, limiting our ability to assess the clinical severity of hyponatremia in symptomatic terms. Second, cancer staging data were unavailable for a significant portion of the cohort, precluding stratified analyses based on disease stage—an important omission given that tumor-induced ADH secretion or organ compromise may alter hyponatremia risk. Third, although hormonal biomarkers, such as cortisol, aldosterone, and ADH, are biologically relevant to sodium regulation, these were not retrievable from our dataset. While we minimized hormonal confounding by balancing hormone-related comorbidities (e.g., diabetes), the lack of direct biomarker data remains a limitation.

This study also faced intrinsic limitations related to its observational design. Despite propensity score matching, residual confounding could not be entirely excluded. Several key laboratory variables—including serum creatinine, LDL, and hemoglobin—remained imbalanced between cohorts, with standardized mean differences exceeding 0.1. These variables may independently influence hyponatremia risk: elevated creatinine reflects impaired renal clearance, low LDL may indicate malnutrition or systemic inflammation, and reduced hemoglobin may reflect volume overload or disease severity. These imbalances may partially bias the observed associations. Additionally, due to TriNetX constraints, only the most recent sodium value per day was accessible, potentially excluding transient sodium fluctuations. Furthermore, environmental exposures such as indoor and outdoor temperature, hydration access, or hospitalization conditions, which may influence hyponatremia risk, were not available in the dataset and could not be adjusted for. Finally, medication dosing and regimen-specific details were unavailable, and our findings may not generalize to cancer types beyond respiratory and intrathoracic malignancies.

## 5. Conclusions

Hyponatremia is a significant risk in respiratory and intrathoracic cancer patients treated with ICI combined with cisplatin, particularly within the first 30 days of treatment. The combination therapy group showed consistently higher cumulative incidences across all sodium thresholds, with mild to moderate hyponatremia being most prominent. Early-phase risk reflects the overlapping effects of ICI-induced immune-related events and cisplatin-induced nephrotoxicity, highlighting the need for vigilant monitoring and personalized interventions. Routine sodium monitoring and tailored management strategies are essential to mitigate risks while maintaining therapeutic efficacy.

## Figures and Tables

**Table 1:** Baseline characteristics of patients in the ICI without and with cisplatin combination groups before and after propensity score matching.

	Before Matching			After Matching		
	ICI with Cisplatin (*n* = 14,782)	ICI Without Cisplatin Combination (*n* = 8076)	Std. Diff.	ICI with Cisplatin (*n* = 7013)	ICI Without Cisplatin Combination (*n* = 7013)	Std. Diff.
Demographics						
Age at index date (mean ± SD)	65.3 ± 9.5	67.0 ± 10.5	0.167	66.7 ± 9.3	66.8 ± 10.4	0.004
Male (%)	52.1%	55.0%	0.059	53.5%	53.6%	0.002
White (%)	67.7%	67.2%	0.009	66.8%	67.2%	0.009
Black or African American (%)	10.0%	9.0%	0.034	10.3%	9.8%	0.018
Comorbidities (%)						
Hypertensive diseases	26.5%	25.4%	0.026	24.5%	25.8%	0.032
Chronic lower respiratory diseases	23.1%	17.8%	0.132	18.6%	19.2%	0.015
Diabetes mellitus	10.2	10.2	0.132	9.5	10.4	0.029
Ischemic heart diseases	9.4	9.5	0.004	9.2	9.6	0.014
Influenza and pneumonia	7.1	6.1	0.041	6.0	6.5	0.021
Malignant neoplasms of skin	2.0	11.0	0.370	4.3	4.6	0.012
Cerebrovascular diseases	3.9	4.1	0.011	4.1	4.1	0.001
Medications (%)						
Blood glucose regulation agents	17.2%	13.2%	0.111	13.2%	13.7%	0.013
Antiarrhythmics	31.1%	23.9%	0.163	24.8%	25.2%	0.009
Beta-blockers	12.6%	13.8%	0.036	13.2%	13.8%	0.019
ACEI	5.3%	6.4%	0.044	5.7%	5.9%	0.011
ARB	4.4%	4.6%	0.007	4.2%	4.6%	0.021
CCB	8.3%	8.3%	<0.001	8.1%	8.4%	0.011
Loop diuretics	6.9%	7.3%	0.015	6.8%	7.3%	0.019
Thiazides diuretics	3.6%	3.4%	0.011	3.0%	3.4%	0.019
Laboratory, Mean ± SD						
Sodium, mmol/L	137.5 ± 3.6	137.7 ± 3.7	0.063	137.5 ± 3.7	137.7 ± 3.8	0.031
Potassium, mmol/L	4.2 ± 0.5	4.2 ± 0.5	0.087	4.2 ± 0.5	4.2 ± 0.5	0.056
Creatinine, mg/dL	0.9 ± 0.4	1.0 ± 0.7	0.141	0.9 ± 0.4	1.0 ± 0.7	0.108
Glucose, mg/dL	119.1 ± 42.0	117.5 ± 42.2	0.037	120.0 ± 43.4	117.8 ± 42.6	0.051
HbA1c, %	6.9 ± 2.1	6.9 ± 2.1	0.013	6.8 ± 1.8	6.9 ± 2.2	0.070
Albumin, g/dL	3.6 ± 0.6	3.6 ± 0.6	0.052	3.6 ± 0.6	3.6 ± 0.6	0.070
LDL, mg/dL	93.6 ± 41.9	86.0 ± 36.1	0.193	91.6 ± 40.3	85.0 ± 37.1	0.171
Hemoglobin, g/dL	11.7 ± 2.0	11.9 ± 2.1	0.129	11.6 ± 2.0	11.8 ± 2.1	0.113

ACEI: angiotensin-converting enzyme inhibitor, ARB: angiotensin II receptor blocker, CCB: calcium channel blocker, HbA1c: hemoglobin A1c, LDL: low-density lipoprotein, SD: standard deviation, Std. Diff.: standardized difference.

## Data Availability

The data presented in this study are available on request due to privacy and ethical restrictions. The dataset was obtained from the TriNetX global federated health research network, and access was subject to institutional approval and data-sharing agreements.

## Supplementary Materials

The following supporting information can be downloaded at: https://www.mdpi.com/article/10.3390/cancers17091459/s1, Supplementary Data S1: Inclusion and exclusion criteria—Detailed logic and temporal constraints used to define cohort eligibility; Supplementary Data S2: STROBE Checklist—Reporting compliance with Strengthening the Reporting of Observational Studies in Epidemiology guidelines; Supplementary Data S3: List of other chemotherapy agents—A comprehensive list of concomitant chemotherapies administered in both treatment cohorts; Supplementary Data S4: Propensity score matching—Baseline characteristics of the study cohorts before and after matching, with statistical comparisons.

## Abbreviations

The following abbreviations are used in this manuscript:

CTLA-4	Cytotoxic T-lymphocyte associated protein 4
eGFR	Directory of open access journals
HbA1c	Hemoglobin A1c
ICI	Immune checkpoint inhibitor
irAEs	Immune-related adverse events
LDL	Low-density lipoprotein
NSCLC	Non-small cell lung cancer
PD-1	Programmed cell death protein 1
PD-L1	Programmed death-ligand 1
RSWS	Renal salt-wasting syndrome
SCLC	Small cell lung cancer
SIADH	Syndrome of inappropriate antidiuretic hormone secretion
