# Supplementary material for: Temporal Trends of Hyponatremia in Patients with Respiratory and Intrathoracic Cancers Treated with Chemotherapy and Immune Checkpoint Inhibitors
Source: Cancers (Basel). 2025 Apr 26;17(9):1459. doi: 10.3390/cancers17091459 (PMC12071158; doi:10.3390/cancers17091459)
Supplement: Supplementary file 1 [file cancers-17-01459-s001.zip › cancers-3568797-supplementary.pdf]

Supplementary Materials

Temporal Trends of Hyponatremia in Patients with Respiratory and Intrathoracic Cancers Treated with Chemotherapy and Immune Checkpoint Inhibitors

Kuo-Cheng Lu, Ching-Liang Ho, Joshua Wang, Cai-Mei Zheng, Kuo-Wang Tsai, Yi-Chou Hou and Chien-Lin Lu

Supplementary Data S1: Inclusion and exclusion criteria—Detailed logic and temporal constraints used to define cohort eligibility

Cohorts definition

This section lists all terms used in the definitions of the two cohorts.

Query Criteria for Cohort 1 (query name: ICI + CiS Lung 1227)

This query was run on the network Global Collaborative Network with 143 HCO(s) queried and 143 HCO(s) responded. A total of 83 provider(s) responded with patients. The final cohort included 17,991 patients who matched the query criteria listed in the table below. For the text representation of the query criteria please see Appendix A.

**Ungrouped terms**				
must have		demographics	Age	Age (at least 18 years (most recent occurrence))
**Group 1**				
**Group 1A Lung Ca**				
must have		diagnosis	UMLS:ICD10CM:C30-C39	Malignant neoplasms of respiratory and intrathoracic organs (at least 18 years old at event)
cannot have		diagnosis	UMLS:ICD10CM:E27.1	Primary adrenocortical insufficiency (at least 18 years old at event)
date constraint	The terms in this group occurred between Jan 1, 2011 and Jan 1, 2021			
event relationship	Any instance of CIS+PEVG occurred within 6 months on or after any instance of Lung Ca			
**Group 1B CIS+PEVG**				
must have	any of	medication	NLM:RXNORM:40048	carboplatin
		medication	NLM:RXNORM:2555	cisplatin
	and any of	medication	NLM:RXNORM:1919503	durvalumab
		medication	NLM:RXNORM:1875534	avelumab
		medication	NLM:RXNORM:1094833	ipilimumab
		medication	NLM:RXNORM:2619313	tremelimumab
		medication	NLM:RXNORM:1547545	pembrolizumab
		medication	NLM:RXNORM:1597876	nivolumab
		medication	NLM:RXNORM:1792776	atezolizumab
		medication	NLM:RXNORM:2058826	cemiplimab

Query Criteria for Cohort 2 (query name: ICI - CiS Lung 1227)

This query was run on the network Global Collaborative Network with 143 HCO(s) queried and 143 HCO(s) responded. A total of 98 provider(s) responded with patients. The final cohort included 9489 patients who matched the query criteria listed in the table below.

**Ungrouped terms**				
must have		demographics	Age	Age (at least 18 years (most recent occurrence))
**Group 1**				
**Group 1A Lung Ca**				
must have		diagnosis	UMLS:ICD10CM:C30-C39	Malignant neoplasms of respiratory and intrathoracic organs (at least 18 years old at event)
cannot have		diagnosis	UMLS:ICD10CM:E27.1	Primary adrenocortical insufficiency (at least 18 years old at event)
date constraint		The terms in this group occurred between Jan 1, 2011 and Jan 1, 2021		
event relationship		Any instance of CIS+PEVG occurred within 6 months on or after any instance of Lung Ca		
**Group 1B CIS+PEVG**				
must have	any of	medication	NLM:RXNORM:1919503	durvalumab
		medication	NLM:RXNORM:1875534	avelumab
		medication	NLM:RXNORM:1094833	ipilimumab
		medication	NLM:RXNORM:2619313	tremelimumab
		medication	NLM:RXNORM:1547545	pembrolizumab
		medication	NLM:RXNORM:1597876	nivolumab
		medication	NLM:RXNORM:1792776	atezolizumab
		medication	NLM:RXNORM:2058826	cemiplimab
cannot have		medication	NLM:RXNORM:40048	carboplatin
	or	medication	NLM:RXNORM:2555	cisplatin

Analysis Setup

This section contains the Index Event and Time Window definitions and a list of selected outcomes and the analyses.

Index Event & Time Window Definitions

The index event defines the point in time when each patient in the cohort enters the analysis. To define an index event for the cohort, one or more criteria for the cohort must be selected. The index date for each patient within a cohort is the day on which the patient first met the selected criteria for the cohort (listed in the table below).

As the index event defines the earliest time point after which outcomes are analyzed, the time window defines the duration during which outcomes are analyzed. The time window can start on the same day as the index event or at any specified time interval after the index event. The time window can end any time after the start date. Outcomes are defined as diagnoses, medications, procedures, or laboratory values that happened in the time window starting after the first occurrence of the index event.

Time Window Used in this Analysis

This analysis included outcomes that occurred in the time window that started 91 days after the first occurrence of the index event and ended 91 days after the first occurrence of the index event.

The index event only includes events that occurred up to 20 years ago. Patients whose index event occurred 20 years or more ago are excluded. In this analysis, 0 patients in Cohort 1 and 0 patients in Cohort 2 were excluded because they met the index event more than 20 years ago.

Index Events Used in this Analysis

Index events for the Compare Outcomes analysis were derived from the cohort definitions. Index events were defined separately for each cohort and were based on the criteria used in the original cohort definition. Please see Appendix B for the text representation of the index event definition.

The index event for Cohort 1 (query name: ICI + CiS Lung 1227) was defined as the following:

**Group 1**				
**Group 1A Lung Ca**				
must have		diagnosis	UMLS:ICD10CM:C30-C39	Malignant neoplasms of respiratory and intrathoracic organs (at least 18 years old at event)
cannot have		diagnosis	UMLS:ICD10CM:E27.1	Primary adrenocortical insufficiency (at least 18 years old at event)
date constraint	The terms in this group occurred between Jan 1, 2011 and Jan 1, 2021			
event relationship	Any instance of CIS+PEVG occurred within 6 months on or after any instance of Lung Ca			
**Group 1B CIS+PEVG**				
must have	any of	medication	NLM:RXNORM:40048	carboplatin
		medication	NLM:RXNORM:2555	cisplatin
	and any of	medication	NLM:RXNORM:1919503	durvalumab
		medication	NLM:RXNORM:1875534	avelumab
		medication	NLM:RXNORM:1094833	ipilimumab
		medication	NLM:RXNORM:2619313	tremelimumab
		medication	NLM:RXNORM:1547545	pembrolizumab
		medication	NLM:RXNORM:1597876	nivolumab
		medication	NLM:RXNORM:1792776	atezolizumab
		medication	NLM:RXNORM:2058826	cemiplimab

The index event for Cohort 2 (query name: ICI - CiS Lung 1227) was defined as the following:

**Group 1**				
**Group 1A Lung Ca**				
must have		diagnosis	UMLS:ICD10CM:C30-C39	Malignant neoplasms of respiratory and intrathoracic organs (at least 18 years old at event)
cannot have		diagnosis	UMLS:ICD10CM:E27.1	Primary adrenocortical insufficiency (at least 18 years old at event)
date constraint	The terms in this group occurred between Jan 1, 2011 and Jan 1, 2021			
event relationship	Any instance of CIS+PEVG occurred within 6 months on or after any instance of Lung Ca			
**Group 1B CIS+PEVG**				
must have	any of	medication	NLM:RXNORM:1919503	durvalumab
		medication	NLM:RXNORM:1875534	avelumab
		medication	NLM:RXNORM:1094833	ipilimumab
		medication	NLM:RXNORM:2619313	tremelimumab
		medication	NLM:RXNORM:1547545	pembrolizumab
		medication	NLM:RXNORM:1597876	nivolumab
		medication	NLM:RXNORM:1792776	atezolizumab
		medication	NLM:RXNORM:2058826	cemiplimab
cannot have		medication	NLM:RXNORM:40048	carboplatin
	or	medication	NLM:RXNORM:2555	cisplatin

Supplementary Data S2: STROBE Checklist—Reporting compliance with Strengthening the Reporting of Observational Studies in Epidemiology guidelines

STROBE Statement—checklist of items that should be included in reports of observational studies

	Item No	Recommendation	Page No
**Title and abstract**	1	(*a*) Indicate the study's design with a commonly used term in the title or the abstract	1
		(*b*) Provide in the abstract an informative and balanced summary of what was done and what was found	2
		**Introduction**	
Background/rationale	2	Explain the scientific background and rationale for the investigation being reported	2
Objectives	3	State specific objectives, including any prespecified hypotheses	3
		**Methods**	
Study design	4	Present key elements of study design early in the paper	5
Setting	5	Describe the setting, locations, and relevant dates, including periods of recruitment, exposure, follow-up, and data collection	5
Participants	6	(*a*) *Cohort study*—Give the eligibility criteria, and the sources and methods of selection of participants. Describe methods of follow-up<br>*Case-control study*—Give the eligibility criteria, and the sources and methods of case ascertainment and control selection. Give the rationale for the choice of cases and controls<br>*Cross-sectional study*—Give the eligibility criteria, and the sources and methods of selection of participants	6
		(*b*) *Cohort study*—For matched studies, give matching criteria and number of exposed and unexposed<br>*Case-control study*—For matched studies, give matching criteria and the number of controls per case	6
Variables	7	Clearly define all outcomes, exposures, predictors, potential confounders, and effect modifiers. Give diagnostic criteria, if applicable	6
Data sources/ measurement	8*	For each variable of interest, give sources of data and details of methods of assessment (measurement). Describe comparability of assessment methods if there is more than one group	*6*
Bias	9	Describe any efforts to address potential sources of bias	6
Study size	10	Explain how the study size was arrived at	5
Quantitative variables	11	Explain how quantitative variables were handled in the analyses. If applicable, describe which groupings were chosen and why	6
Statistical methods	12	(*a*) Describe all statistical methods, including those used to control for confounding	6-7
		(*b*) Describe any methods used to examine subgroups and interactions	6-7
		(*c*) Explain how missing data were addressed	6-7
		(*d*) *Cohort study*—If applicable, explain how loss to follow-up was addressed<br>*Case-control study*—If applicable, explain how matching of cases and controls was addressed<br>*Cross-sectional study*—If applicable, describe analytical methods taking account of sampling strategy	6-7
		(*e*) Describe any sensitivity analyses	6-7

Continued on next page

**Results**			
Participants	13*	(a) Report numbers of individuals at each stage of study—eg numbers potentially eligible, examined for eligibility, confirmed eligible, included in the study, completing follow-up, and analysed	4-5
		(b) Give reasons for non-participation at each stage	4-5
		(c) Consider use of a flow diagram	4-5
Descriptive data	14*	(a) Give characteristics of study participants (eg demographic, clinical, social) and information on exposures and potential confounders	7-9
		(b) Indicate number of participants with missing data for each variable of interest	7-9
		(c) *Cohort study*—Summarise follow-up time (eg, average and total amount)	7
Outcome data	15*	*Cohort study*—Report numbers of outcome events or summary measures over time	*Table 1, 7-9*
		Case-control study—Report numbers in each exposure category, or summary measures of exposure	*n/a*
		Cross-sectional study—Report numbers of outcome events or summary measures	*n/a*
Main results	16	(*a*) Give unadjusted estimates and, if applicable, confounder-adjusted estimates and their precision (eg, 95% confidence interval). Make clear which confounders were adjusted for and why they were included	7-9
		(*b*) Report category boundaries when continuous variables were categorized	7-9
		(*c*) If relevant, consider translating estimates of relative risk into absolute risk for a meaningful time period	7-9
Other analyses	17	Report other analyses done—eg analyses of subgroups and interactions, and sensitivity analyses	7-9
**Discussion**			
Key results	18	Summarise key results with reference to study objectives	10-12
Limitations	19	Discuss limitations of the study, taking into account sources of potential bias or imprecision. Discuss both direction and magnitude of any potential bias	12
Interpretation	20	Give a cautious overall interpretation of results considering objectives, limitations, multiplicity of analyses, results from similar studies, and other relevant evidence	12
Generalisability	21	Discuss the generalisability (external validity) of the study results	12
**Other information**			
Funding	22	Give the source of funding and the role of the funders for the present study and, if applicable, for the original study on which the present article is based	13

*Give information separately for cases and controls in case-control studies and, if applicable, for exposed and unexposed groups in cohort and cross-sectional studies.

Note: An Explanation and Elaboration article discusses each checklist item and gives methodological background and published examples of transparent reporting. The STROBE checklist is best used in conjunction with this article (freely available on the Web sites of PLoS Medicine at http://www.plosmedicine.org/, Annals of Internal Medicine at http://www.annals.org/, and Epidemiology at http://www.epidem.com/). Information on the STROBE Initiative is available at www.strobe-statement.org (accessed on 1 January 2020).

Supplementary 3. List of Other Chemotherapy Agents Administered Alongside ICIs or ICIs with Cisplatin/Carboplatin in Study Cohorts

ICI + CIS

VAAN900	Antineoplastic, other	16,574	100%
RxNorm40048	Carboplatin	15,029	91%
RxNorm1547545	Pembrolizumab	7982	48%
RxNorm56946	Paclitaxel	6969	42%
RxNorm4179	Etoposide	3411	21%
RxNorm2555	Cisplatin	3333	20%
RxNorm72962	Docetaxel	2632	16%
RxNorm12574	Gemcitabine	2591	16%

RxNorm1919503	Durvalumab	2473	15%
RxNorm1792776	Atezolizumab	2434	15%
RxNorm253337	Bevacizumab	1406	8%
RxNorm39541	Vinorelbine	767	5%
RxNorm337525	Erlotinib	483	3%
RxNorm318341	Cetuximab	446	3%
RxNorm1721560	Osimertinib	443	3%
RxNorm6851	Methotrexate	281	2%
RxNorm194000	Capecitabine	138	1%
VAAN300	Antimetabolites	10,909	66%
RxNorm68446	Pemetrexed	7520	45%
RxNorm4492	Fluorouracil	618	4%
RxNorm6851	Methotrexate	281	2%
RxNorm194000	Capecitabine	138	1%

ICI – CIS

VAAN900	Antineoplastic, other	6493	99%
RxNorm1547545	Pembrolizumab	3646	56%
RxNorm56946	Paclitaxel	617	9%
RxNorm40048	Carboplatin	496	8%
RxNorm12574	Gemcitabine	445	7%
RxNorm72962	Docetaxel	437	7%
RxNorm1792776	Atezolizumab	376	6%
RxNorm1919503	Durvalumab	295	5%
RxNorm253337	Bevacizumab	281	4%
RxNorm2555	Cisplatin	280	4%
RxNorm1363268	Cabozantinib	180	3%
VAAN300	Antineoplastics,antimetabolites	3197	49%
RxNorm68446	Pemetrexed	611	9%
RxNorm4492	Fluorouracil	305	5%
RxNorm6851	Methotrexate	122	2%
RxNorm194000	Capecitabine	90	1%

Supplementary Data S4: Propensity score matching—Baseline characteristics of the study cohorts before and after matching, with statistical comparisons.

Propensity Score Matching

Propensity score matching was performed on all listed characteristics. Characteristics of the cohorts before and after matching are summarized in the table below.

Cohort 1 and cohort 2 patient count before and after propensity score matching

Cohort		Patient count before matching	Patient count after matching
1 - ICI + CiS	Lung 1227	14,782	7013
2 - ICI - CiS	Lung 1227	8076	7013

Propensity score density function - Before and after matching (cohort 1 - purple, cohort 2 - green)

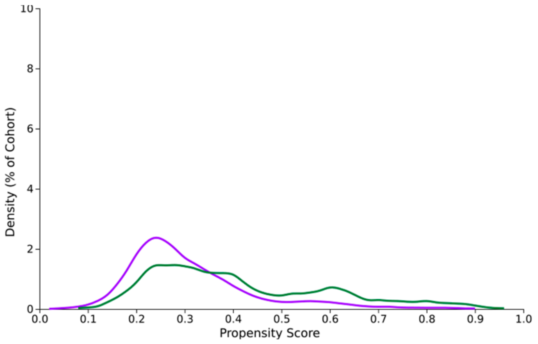

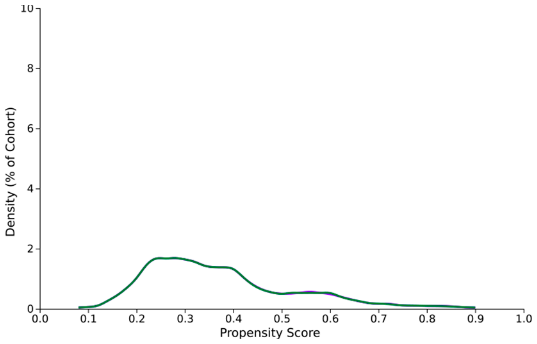

Cohort 1 (N = 14,782) and cohort 2 (N = 8076) characteristics before propensity score matching

			Mean ± SD	Patients	% of Cohort	P-Value	Std diff.
**Demographics**							
Cohort			Mean ± SD	Patients	% of Cohort	P-Value	Std diff.
1<br>2	AI	Age at Index	65.3 ± 9.5<br>67.0 ± 10.5	14,782<br>8076	100%<br>100%	<0.001	0.167
1<br>2	2106-3	White		10,003<br>5430	67.7%<br>67.2%	0.503	0.009
1<br>2	F	Female		6158<br>3267	41.7%<br>40.5%	0.077	0.025
1<br>2	2054-5	Black or African American		1473<br>725	10.0%<br>9.0%	0.015	0.034
1<br>2	M	Male		7697<br>4442	52.1%<br>55.0%	<0.001	0.059
1<br>2	2028-9	Asian		876<br>286	5.9%<br>3.5%	<0.001	0.112
**Diagnosis**							
Cohort			Mean ± SD	Patients	% of Cohort	P-Value	Std diff.
1<br>2	C30-C39	Malignant neoplasms of respiratory and intrathoracic organs		13,298<br>6102	90.0%<br>75.6%	<0.001	0.388
1<br>2	C43-C44	Melanoma and other malignant neoplasms of skin		303<br>892	2.0%<br>11.0%	<0.001	0.370
1<br>2	I10-I1A	Hypertensive diseases		3919<br>2049	26.5%<br>25.4%	0.061	0.026
1<br>2	I20-I25	Ischemic heart diseases		1392<br>769	9.4%<br>9.5%	0.795	0.004
1<br>2	I60-I69	Cerebrovascular diseases		571<br>330	3.9%<br>4.1%	0.407	0.011
1<br>2	J09-J18	Influenza and pneumonia		1050<br>492	7.1%<br>6.1%	0.004	0.041
1<br>2	J40-J4A	Chronic lower respiratory diseases		3417<br>1438	23.1%<br>17.8%	<0.001	0.132
1<br>2	E08-E13	Diabetes mellitus		1509<br>826	10.2%<br>10.2%	0.963	0.001
**Medication**							
Cohort			Mean ± SD	Patients	% of Cohort	P-Value	Std diff.
1<br>2	HS500	BLOOD GLUCOSE REGULATION AGENTS		2546<br>1070	17.2%<br>13.2%	<0.001	0.111
1<br>2	CV300	ANTIARRHYTHMICS		4603<br>1929	31.1%<br>23.9%	<0.001	0.163
1<br>2	CV100	BETA BLOCKERS/RELATED		1865<br>1117	12.6%<br>13.8%	0.009	0.036
1<br>2	CV700	DIURETICS		1597<br>871	10.8%<br>10.8%	0.965	0.001
1<br>2	CV200	CALCIUM CHANNEL BLOCKERS		1230<br>671	8.3%<br>8.3%	0.974	<0.001
1<br>2	CV800	ACE INHIBITORS		790<br>516	5.3%<br>6.4%	0.001	0.044
1<br>2	CV805	ANGIOTENSIN II INHIBITOR		657<br>370	4.4%<br>4.6%	0.633	0.007
1<br>2	CV702	LOOP DIURETICS		1022<br>590	6.9%<br>7.3%	0.269	0.015

Cohort			Mean ± SD	Patients	% of Cohort	P-Value	Std diff.
1<br>2	CV701	THIAZIDES/RELATED DIURETICS		538<br>278	3.6%<br>3.4%	0.442	0.011
**Laboratory**							
Cohort			Mean ± SD	Patients	% of Cohort	P-Value	Std diff.
1<br>2	9029	Sodium [Moles/volume] in Serum, Plasma or Blood	137.5 ± 3.6<br>137.7 ±3.7	10,499<br>4927	71.0%<br>61.0%	<0.001	0.063
1<br>2		0 - 0 mmol/L		10,500<br>4927	71.0%<br>61.0%	<0.001	0.213
1<br>2	9028	Potassium [Moles/volume] in Serum, Plasma or Blood	4.2 ± 0.5<br>4.2 ± 0.5	10,518<br>4951	71.2%<br>61.3%	<0.001	0.087
1<br>2		0 - 0 mmol/L		10,518<br>4951	71.2%<br>61.3%	<0.001	0.209
1<br>2	9030	Urea nitrogen [Mass/volume] in Serum, Plasma or Blood	16.0 ± 7.4<br>17.7 ± 9.5	9347<br>4119	63.2%<br>51.0%	<0.001	0.198
1<br>2		0 - 0 mg/dL		9348<br>4119	63.2%<br>51.0%	<0.001	0.249
1<br>2	9024	Creatinine [Mass/volume] in Serum, Plasma or Blood	0.9 ± 0.4<br>1.0 ± 0.7	10,625<br>4718	71.9%<br>58.4%	<0.001	0.141
1<br>2		0 - 0 mg/dL		10,625<br>4718	71.9%<br>58.4%	<0.001	0.285
1<br>2	9022	Calcium [Mass/volume] in Serum, Plasma or Blood	9.3 ± 0.6<br>9.2 ± 0.7	10,146<br>4512	68.6%<br>55.9%	0.058	0.034
1<br>2		0 - 0 mg/dL		10,146<br>4512	68.6%<br>55.9%	<0.001	0.266
1<br>2	9026	Magnesium [Mass/volume] in Serum, Plasma or Blood	1.9 ± 0.3<br>1.9 ± 0.3	4179<br>1810	28.3%<br>22.4%	<0.001	0.128
1<br>2		0 - 0 mg/dL		4179<br>1810	28.3%<br>22.4%	<0.001	0.135
1<br>2	9025	Glucose [Mass/volume] in Serum, Plasma or Blood	119.1 ± 42.0<br>117.5 ± 42.2	10,196<br>4902	69.0%<br>60.7%	0.034	0.037
1<br>2		0 - 0 mg/dL		10,196<br>4902	69.0%<br>60.7%	<0.001	0.174
1<br>2	9014	Hemoglobin [Mass/volume] in Blood	11.7 ± 2.0<br>11.9 ± 2.1	10,662<br>4960	72.1%<br>61.4%	<0.001	0.129
1<br>2		0 - 0 g/dL		10,662<br>4960	72.1%<br>61.4%	<0.001	0.229
1<br>2	9013	Hematocrit [Volume Fraction] of Blood	35.1 ± 7.2<br>36.1 ± 7.0	10,615<br>5012	71.8%<br>62.1%	<0.001	0.146
1<br>2		0 - 0 %		10,615<br>5013	71.8%<br>62.1%	<0.001	0.208
1<br>2	9015	Leukocytes [#/volume] in Blood	64.2 ± 467.4<br>17.2 ± 186.6	9844<br>4583	66.6%<br>56.7%	<0.001	0.132
1<br>2		0 - 0 10*3/uL		10,077<br>4620	68.2%<br>57.2%	<0.001	0.228
1<br>2	9044	Alanine aminotransferase [Enzymatic activity/volume] in Serum, Plasma or Blood	25.2 ± 28.3<br>25.2 ± 33.4	10,043<br>4607	67.9%<br>57.0%	0.911	0.002
1<br>2		0 - 0 U/L		10,043<br>4607	67.9%<br>57.0%	<0.001	0.226

1<br>2	9047	Aspartate aminotransferase [Enzymatic activity/volume] in Serum or Plasma	25.9 ± 26.2<br>26.7 ± 34.1	9962<br>4596	67.4%<br>56.9%	0.103	0.028
1<br>2		0 - 0 U/L		9962<br>4596	67.4%<br>56.9%	<0.001	0.217
1<br>2	9046	Alkaline phosphatase [Enzymatic activity/volume] in Serum, Plasma or Blood	111.8 ± 94.8<br>113.4 ± 142.0	9864<br>4482	66.7%<br>55.5%	0.427	0.013
1<br>2		0 - 0 U/L		9864<br>4482	66.7%<br>55.5%	<0.001	0.232
1<br>2	9045	Albumin [Mass/volume] in Serum, Plasma or Blood	3.6 ± 0.6<br>3.6 ± 0.6	9406<br>4331	63.6%<br>53.6%	0.004	0.052
1<br>2		0 - 0 g/dL		9410<br>4333	63.7%<br>53.7%	<0.001	0.204
1<br>2	9002	Cholesterol in LDL [Mass/volume] in Serum or Plasma	93.6 ± 41.9<br>86.0 ± 36.1	487<br>261	3.3%<br>3.2%	0.014	0.193
1<br>2		0 - 0 mg/dL		487<br>261	3.3%<br>3.2%	0.799	0.004
1<br>2	9004	Triglyceride [Mass/volume] in Serum, Plasma or Blood	125.6 ± 78.3<br>130.6 ± 84.1	588<br>333	4.0%<br>4.1%	0.366	0.061
1<br>2		0 - 0 mg/dL		588<br>333	4.0%<br>4.1%	0.593	0.007
1<br>2	9037	Hemoglobin A1c/Hemoglobin.total in Blood	6.9 ± 2.1<br>6.9 ± 2.1	655<br>321	4.4%<br>4.0%	0.849	0.013
1<br>2		0 - 0 %		655<br>321	4.4%<br>4.0%	0.103	0.023
1<br>2	9039	Parathyrin.intact [Mass/volume] in Serum or Plasma	55.4 ± 106.2<br>62.9 ± 121.1	107<br>67	0.7%<br>0.8%	0.669	0.066
1<br>2		0 - 0 pg/mL		107<br>67	0.7%<br>0.8%	0.379	0.012
1<br>2	9034	Calcidiol [Mass/volume] in Serum or Plasma	29.8 ± 16.1<br>27.5 ± 15.0	124<br>63	0.8%<br>0.8%	0.356	0.145
1<br>2		0 - 0 ng/mL		124<br>63	0.8%<br>0.8%	0.637	0.007
1<br>2	9063	C reactive protein [Mass/volume] in Serum, Plasma or Blood	49.4 ± 66.8<br>55.4 ± 69.3	771<br>623	5.2%<br>7.7%	0.104	0.087
1<br>2		0 - 0 mg/L		771<br>623	5.2%<br>7.7%	<0.001	0.102
1<br>2	9066	Erythrocyte sedimentation rate	54.6 ± 35.6<br>47.9 ± 36.1	248<br>147	1.7%<br>1.8%	0.073	0.187
1<br>2		0 - 0 mm/h		248<br>147	1.7%<br>1.8%	0.429	0.011
1<br>2	9000	Cholesterol [Mass/volume] in Serum or Plasma	164.6 ± 51.0<br>155.9 ± 48.2	561<br>324	3.8%<br>4.0%	0.013	0.176
1<br>2		0 - 0 mg/dL		561<br>324	3.8%<br>4.0%	0.417	0.011

Cohort 1 (N = 7013) and cohort 2 (N = 7013) characteristics after propensity score matching

Cohort			Mean ± SD	Patients	% of Cohort	P-Value	Std diff.
**Demographics**							
1<br>2	AI	Age at Index	66.7 ± 9.3<br>66.8 ± 10.4	7013<br>7013	100%<br>100%	0.824	0.004
1<br>2	2106-3	White		4685<br>4715	66.8%<br>67.2%	0.590	0.009
1<br>2	F	Female		2934<br>2908	41.8%<br>41.5%	0.656	0.008
1<br>2	2054-5	Black or African American		723<br>685	10.3%<br>9.8%	0.286	0.018
1<br>2	M	Male		3755<br>3762	53.5%<br>53.6%	0.906	0.002
1<br>2	2028-9	Asian		241<br>275	3.4%<br>3.9%	0.127	0.026
**Diagnosis**							
Cohort			Mean ± SD	Patients	% of Cohort	P-Value	Std diff.
1<br>2	C30-C39	Malignant neoplasms of respiratory and intrathoracic organs		5650<br>5669	80.6%<br>80.8%	0.684	0.007
1<br>2	C43-C44	Melanoma and other malignant neoplasms of skin		303<br>320	4.3%<br>4.6%	0.486	0.012
1<br>2	I10-I1A	Hypertensive diseases		1716<br>1812	24.5%<br>25.8%	0.062	0.032
1<br>2	I20-I25	Ischemic heart diseases		646<br>674	9.2%<br>9.6%	0.418	0.014
1<br>2	I60-I69	Cerebrovascular diseases		287<br>285	4.1%<br>4.1%	0.932	0.001
1<br>2	J09-J18	Influenza and pneumonia		424<br>459	6.0%<br>6.5%	0.224	0.021
1<br>2	J40-J4A	Chronic lower respiratory diseases		1304<br>1345	18.6%<br>19.2%	0.376	0.015
1<br>2	E08-E13	Diabetes mellitus		666<br>727	9.5%<br>10.4%	0.085	0.029
**Medication**							
Cohort			Mean ± SD	Patients	% of Cohort	P-Value	Std diff.
1<br>2	HS500	BLOOD GLUCOSE REGULATION AGENTS		929<br>961	13.2%<br>13.7%	0.429	0.013
1<br>2	CV300	ANTIARRHYTHMICS		1738<br>1766	24.8%<br>25.2%	0.585	0.009
1<br>2	CV100	BETA BLOCKERS/RELATED		923<br>968	13.2%<br>13.8%	0.266	0.019
1<br>2	CV700	DIURETICS		708<br>759	10.1%<br>10.8%	0.159	0.024
1<br>2	CV200	CALCIUM CHANNEL BLOCKERS		569<br>591	8.1%<br>8.4%	0.500	0.011
1<br>2	CV800	ACE INHIBITORS		398<br>416	5.7%<br>5.9%	0.516	0.011
1<br>2	CV805	ANGIOTENSIN II INHIBITOR		296<br>326	4.2%<br>4.6%	0.219	0.021
1<br>2	CV702	LOOP DIURETICS		480<br>515	6.8%<br>7.3%	0.250	0.019

1<br>2	CV701	THIAZIDES/RELATED DIURETICS		212<br>236	3.0%<br>3.4%	0.249	0.019
		**Laboratory**					
Cohort			Mean ± SD	Patients	% of Cohort	P-Value	Std diff.
1<br>2	9029	Sodium [Moles/volume] in Serum, Plasma or Blood	137.5 ± 3.7<br>137.7 ± 3.8	4279<br>4336	61.0%<br>61.8%	0.147	0.031
1<br>2		0 - 0 mmol/L		4280<br>4336	61.0%<br>61.8%	0.331	0.016
1<br>2	9028	Potassium [Moles/volume] in Serum, Plasma or Blood	4.2 ± 0.5<br>4.2 ± 0.5	4294<br>4356	61.2%<br>62.1%	0.009	0.056
1<br>2		0 - 0 mmol/L		4294<br>4356	61.2%<br>62.1%	0.282	0.018
1<br>2	9030	Urea nitrogen [Mass/volume] in Serum, Plasma or Blood	16.4 ± 7.6<br>17.7 ± 9.7	3647<br>3689	52.0%<br>52.6%	<0.001	0.147
1<br>2		0 - 0 mg/dL		3648<br>3689	52.0%<br>52.6%	0.488	0.012
1<br>2	9024	Creatinine [Mass/volume] in Serum, Plasma or Blood	0.9 ± 0.4<br>1.0 ± 0.7	4227<br>4260	60.3%<br>60.7%	<0.001	0.108
1<br>2		0 - 0 mg/dL		4227<br>4260	60.3%<br>60.7%	0.569	0.010
1<br>2	9022	Calcium [Mass/volume] in Serum, Plasma or Blood	9.3 ± 0.7<br>9.2 ± 0.7	4017<br>4063	57.3%<br>57.9%	0.028	0.049
1<br>2		0 - 0 mg/dL		4017<br>4063	57.3%<br>57.9%	0.432	0.013
1<br>2	9026	Magnesium [Mass/volume] in Serum, Plasma or Blood	1.9 ± 0.3<br>1.9 ± 0.3	1633<br>1656	23.3%<br>23.6%	<0.001	0.172
1<br>2		0 - 0 mg/dL		1633<br>1656	23.3%<br>23.6%	0.647	0.008
1<br>2	9025	Glucose [Mass/volume] in Serum, Plasma or Blood	120.0 ± 43.4<br>117.8 ± 42.6	4272<br>4320	60.9%<br>61.6%	0.018	0.051
1<br>2		0 - 0 mg/dL		4272<br>4320	60.9%<br>61.6%	0.405	0.014
1<br>2	9014	Hemoglobin [Mass/volume] in Blood	11.6 ± 2.0<br>11.8 ± 2.1	4309<br>4372	61.4%<br>62.3%	<0.001	0.113
1<br>2		0 - 0 g/dL		4309<br>4372	61.4%<br>62.3%	0.273	0.018
1<br>2	9013	Hematocrit [Volume Fraction] of Blood	34.8 ± 7.5<br>36.0 ± 6.9	4364<br>4410	62.2%<br>62.9%	<0.001	0.159
1<br>2		0 - 0 %		4364<br>4411	62.2%<br>62.9%	0.412	0.014
1<br>2	9015	Leukocytes [#/volume] in Blood	26.5 ± 252.0<br>17.4 ± 188.8	3961<br>4055	56.5%<br>57.8%	0.066	0.041
1<br>2		0 - 0 10*3/uL		4002<br>4089	57.1%<br>58.3%	0.137	0.025
1<br>2	9044	Alanine aminotransferase [Enzymatic activity/volume] in Serum, Plasma or Blood	24.9 ± 28.3<br>25.2 ± 34.5	3994<br>4072	57.0%<br>58.1%	0.736	0.008
1<br>2		0 - 0 U/L		3994<br>4072	57.0%<br>58.1%	0.183	0.023

1<br>2	9047	Aspartate aminotransferase [Enzymatic activity/volume] in Serum or Plasma	25.7 ± 26.9<br>26.7 ± 35.4	3979<br>4051	56.7%<br>57.8%	0.168	0.031
1<br>2		0 - 0 U/L		3979<br>4051	56.7%<br>57.8%	0.219	0.021
1<br>2	9046	Alkaline phosphatase [Enzymatic activity/volume] in Serum, Plasma or Blood	110.6 ± 99.4<br>115.0 ± 148.2	3920<br>3977	55.9%<br>56.7%	0.122	0.035
1<br>2		0 - 0 U/L		3920<br>3977	55.9%<br>56.7%	0.332	0.016
1<br>2	9045	Albumin [Mass/volume] in Serum, Plasma or Blood	3.6 ± 0.6<br>3.6 ± 0.6	3779<br>3841	53.9%<br>54.8%	0.002	0.070
1<br>2		0 - 0 g/dL		3782<br>3843	53.9%<br>54.8%	0.301	0.017
1<br>2	9002	Cholesterol in LDL [Mass/volume] in Serum or Plasma	91.6 ± 40.3<br>85.0 ± 37.1	203<br>227	2.9%<br>3.2%	0.076	0.171
1<br>2		0 - 0 mg/dL		203<br>227	2.9%<br>3.2%	0.240	0.020
1<br>2	9004	Triglyceride [Mass/volume] in Serum, Plasma or Blood	127.7 ± 83.5<br>129.8 ± 84.4	262<br>293	3.7%<br>4.2%	0.762	0.026
1<br>2		0 - 0 mg/dL		262<br>293	3.7%<br>4.2%	0.179	0.023
1<br>2	9037	Hemoglobin A1c/Hemoglobin.total in Blood	6.8 ± 1.8<br>6.9 ± 2.2	274<br>290	3.9%<br>4.1%	0.407	0.070
1<br>2		0 - 0 %		274<br>290	3.9%<br>4.1%	0.492	0.012
1<br>2	9039	Parathyrin.intact [Mass/volume] in Serum or Plasma	58.7 ± 120.9<br>59.8 ± 124.4	52<br>60	0.7%<br>0.9%	0.961	0.009
1<br>2		0 - 0 pg/mL		52<br>60	0.7%<br>0.9%	0.448	0.013
1<br>2	9034	Calcidiol [Mass/volume] in Serum or Plasma	31.4 ± 17.7<br>26.9 ± 14.8	63<br>56	0.9%<br>0.8%	0.135	0.278
1<br>2		0 - 0 ng/mL		63<br>56	0.9%<br>0.8%	0.519	0.011
1<br>2	9063	C reactive protein [Mass/volume] in Serum, Plasma or Blood	52.4 ± 67.1<br>58.4 ± 72.9	427<br>455	6.1%<br>6.5%	0.204	0.086
1<br>2		0 - 0 mg/L		427<br>455	6.1%<br>6.5%	0.330	0.016
1<br>2	9066	Erythrocyte sedimentation rate	57.1 ± 34.1<br>49.6 ± 37.0	114<br>122	1.6%<br>1.7%	0.107	0.211
1<br>2		0 - 0 mm/h		114<br>122	1.6%<br>1.7%	0.599	0.009
1<br>2	9000	Cholesterol [Mass/volume] in Serum or Plasma	162.3 ± 48.3<br>155.0 ± 49.5	260<br>284	3.7%<br>4.0%	0.082	0.150
1<br>2		0 - 0 mg/dL		260<br>284	3.7%<br>4.0%	0.294	0.018

Appendix A – Text Representation of the Cohorts Definition

This section lists all terms used in the definitions of the two cohorts.

Query Criteria for Cohort 1 (query name: 6 個月 -Cis ICIx3 Lung + ICI 1225)

Patients must have:
Age (Age) (at least 18 years (most recent occurrence)).

All the following must be satisfied:

Lung Ca: The terms in this group occurred between Jan 1, 2011 and Jan 1, 2021
Patients must have:
Malignant neoplasms of respiratory and intrathoracic organs (UMLS:ICD10CM:C30-C39) (at least 18 years old at event).
Patients cannot have:
Primary adrenocortical insufficiency (UMLS:ICD10CM:E27.1) (at least 18 years old at event).
ICIs: Any instance of ICIs occurred within 6 months on or after any instance of Lung Ca
Patients must have:
any of the following:
pembrolizumab (NLM:RXNORM:1547545); or
nivolumab (NLM:RXNORM:1597876); or
cemiplimab (NLM:RXNORM:2058826); or
atezolizumab (NLM:RXNORM:1792776); or
avelumab (NLM:RXNORM:1875534); or
durvalumab (NLM:RXNORM:1919503); or
ipilimumab (NLM:RXNORM:1094833); or
tremelimumab (NLM:RXNORM:2619313).
Patients cannot have:
any of the following:
carboplatin (NLM:RXNORM:40048); or
cisplatin (NLM:RXNORM:2555).

ICIs: The terms in this group occurred at any time (Greater than or equal to 3 instances)
Patients must have:
any of the following:
pembrolizumab (NLM:RXNORM:1547545); or
nivolumab (NLM:RXNORM:1597876); or
cemiplimab (NLM:RXNORM:2058826); or
avelumab (NLM:RXNORM:1875534); or
durvalumab (NLM:RXNORM:1919503); or
ipilimumab (NLM:RXNORM:1094833); or
atezolizumab (NLM:RXNORM:1792776); or
tremelimumab (NLM:RXNORM:2619313).

Query Criteria for Cohort 2 (query name: 6 個月 -ICIs CiSx3 Lung + CiS 1225)

Patients must have:
Age (Age) (at least 18 years (most recent occurrence)).

All the following must be satisfied:

Lung Ca: The terms in this group occurred between Jan 1, 2011 and Jan 1, 2021
Patients must have:
Malignant neoplasms of respiratory and intrathoracic organs (UMLS:ICD10CM:C30-C39) (at least 18 years old at event).
Patients cannot have:
Primary adrenocortical insufficiency (UMLS:ICD10CM:E27.1) (at least 18 years old at event).
CIS-ICI: Any instance of CIS-ICI occurred within 6 months on or after any instance of Lung Ca
Patients must have:
any of the following:
carboplatin (NLM:RXNORM:40048); or
cisplatin (NLM:RXNORM:2555).
Patients cannot have:
any of the following:
tremelimumab (NLM:RXNORM:2619313); or
nivolumab (NLM:RXNORM:1597876); or
pembrolizumab (NLM:RXNORM:1547545); or
cemiplimab (NLM:RXNORM:2058826); or
atezolizumab (NLM:RXNORM:1792776); or
ipilimumab (NLM:RXNORM:1094833); or
avelumab (NLM:RXNORM:1875534); or
durvalumab (NLM:RXNORM:1919503).

CISs: The terms in this group occurred at any time (Greater than or equal to 3 instances)
Patients must have:
any of the following:
cisplatin (NLM:RXNORM:2555); or
carboplatin (NLM:RXNORM:40048).

Appendix B – Text Representation of the Analysis Setup

This section contains the Index Event definition for each cohort.

The index event for Cohort 1 (query name: 6 個月 -Cis ICIx3 Lung + ICI 1225) is defined as the following:

All the following must be satisfied:

Lung Ca: The terms in this group occurred between Jan 1, 2011 and Jan 1, 2021
Patients must have:
Malignant neoplasms of respiratory and intrathoracic organs (UMLS:ICD10CM:C30-C39) (at least 18 years old at event).
Patients cannot have:
Primary adrenocortical insufficiency (UMLS:ICD10CM:E27.1) (at least 18 years old at event).
ICIs: Any instance of ICIs occurred within 6 months on or after any instance of Lung Ca
Patients must have:
any of the following:

pembrolizumab (NLM:RXNORM:1547545); or
nivolumab (NLM:RXNORM:1597876); or
cemiplimab (NLM:RXNORM:2058826); or
atezolizumab (NLM:RXNORM:1792776); or
avelumab (NLM:RXNORM:1875534); or
durvalumab (NLM:RXNORM:1919503); or
ipilimumab (NLM:RXNORM:1094833); or
tremelimumab (NLM:RXNORM:2619313).

Patients cannot have:

any of the following:

carboplatin (NLM:RXNORM:40048); or
cisplatin (NLM:RXNORM:2555).

ICIs: The terms in this group occurred at any time (Greater than or equal to 3 instances)

Patients must have:

any of the following:

pembrolizumab (NLM:RXNORM:1547545); or
nivolumab (NLM:RXNORM:1597876); or
cemiplimab (NLM:RXNORM:2058826); or
avelumab (NLM:RXNORM:1875534); or
durvalumab (NLM:RXNORM:1919503); or
ipilimumab (NLM:RXNORM:1094833); or
atezolizumab (NLM:RXNORM:1792776); or
tremelimumab (NLM:RXNORM:2619313).

The index event for Cohort 2 (query name: 6 個月 -ICIs CiSx3 Lung + CiS 1225) is defined as the following:

All the following must be satisfied:

Lung Ca: The terms in this group occurred between Jan 1, 2011 and Jan 1, 2021

Patients must have:

Malignant neoplasms of respiratory and intrathoracic organs (UMLS:ICD10CM:C30-C39) (at least 18 years old at event).

Patients cannot have:

Primary adrenocortical insufficiency (UMLS:ICD10CM:E27.1) (at least 18 years old at event).

CIS-ICI: Any instance of CIS-ICI occurred within 6 months on or after any instance of Lung Ca

Patients must have:

any of the following:

carboplatin (NLM:RXNORM:40048); or
cisplatin (NLM:RXNORM:2555).

Patients cannot have:

any of the following:

tremelimumab (NLM:RXNORM:2619313); or
nivolumab (NLM:RXNORM:1597876); or
pembrolizumab (NLM:RXNORM:1547545); or
cemiplimab (NLM:RXNORM:2058826); or
atezolizumab (NLM:RXNORM:1792776); or
ipilimumab (NLM:RXNORM:1094833); or

avelumab (NLM:RXNORM:1875534); or
durvalumab (NLM:RXNORM:1919503).

CISs: The terms in this group occurred at any time (Greater than or equal to 3 instances)
Patients must have:
any of the following:
cisplatin (NLM:RXNORM:2555); or
carboplatin (NLM:RXNORM:40048).
